# Oxygen Partial Pressure Is a Rate-Limiting Parameter for Cell Proliferation in 3D Spheroids Grown in Physioxic Culture Condition

**DOI:** 10.1371/journal.pone.0161239

**Published:** 2016-08-30

**Authors:** Aurélie Gomes, Ludivine Guillaume, David Robert Grimes, Jérôme Fehrenbach, Valérie Lobjois, Bernard Ducommun

**Affiliations:** 1 ITAV, Université de Toulouse, CNRS, UPS, Toulouse, France; 2 University of Oxford, Oxford, United Kingdom; 3 IMT, Université de Toulouse, CNRS, UPS, Toulouse, France; 4 CHU de Toulouse; Toulouse, France; Medical College of Wisconsin, UNITED STATES

## Abstract

The in situ oxygen partial pressure in normal and tumor tissues is in the range of a few percent. Therefore, when studying cell growth in 3D culture systems, it is essential to consider how the physiological oxygen concentration, rather than the one in the ambient air, influences the proliferation parameters. Here, we investigated the effect of reducing oxygen partial pressure from 21% to 5% on cell proliferation rate and regionalization in a 3D tumor spheroid model. We found that 5% oxygen concentration strongly inhibited spheroid growth, changed the proliferation gradient and reduced the 50% In Depth Proliferation index (IDP_50_), compared with culture at 21% oxygen. We then modeled the oxygen partial pressure profiles using the experimental data generated by culturing spheroids in physioxic and normoxic conditions. Although hypoxia occurred at similar depth in spheroids grown in the two conditions, oxygen partial pressure was a major rate-limiting factor with a critical effect on cell proliferation rate and regionalization only in spheroids grown in physioxic condition and not in spheroids grown at atmospheric normoxia. Our findings strengthen the need to consider conducting experiment in physioxic conditions (i.e., tissue normoxia) for proper understanding of cancer cell biology and the evaluation of anticancer drugs in 3D culture systems.

## Introduction

Eukaryotic cells are dependent on oxygen. Hypoxia describes a condition in which oxygen partial pressure falls below the level necessary to sustain mitochondrial oxidative metabolism and life.

In physiological conditions, oxygen partial pressure in human tissues varies according to their function and needs. As reviewed in [1, 2], while oxygen concentration in air is 21%, its concentration in arterial blood is lower than 14% and definitely lower in tissues (between 14% and 4%), for instance 4.4% in brain, 5.4% in liver and 9.5% in kidney. The lower oxygen partial pressure values observed in tissues, compared to air, do not correspond to hypoxia, but to the tissue physiological condition to maintain its homeostasis and should be called physioxia [2]. Hence, physioxia is the oxygen partial pressure in tissues that defines a threshold ensuring proper physiological function [2]. For instance, it is assumed that oxygen partial pressure above 35mmHg (4.6%) is sufficient to ensure normal oxygenation of the brain tissue. Moreover, results from various studies clearly indicate that tumor hypoxia corresponds to an oxygen partial pressure that is lower relative to the physioxia of the respective normal tissue and not relative to atmospheric normoxia [3, 4]. Genuine hypoxia in tissues and tumors is usually detected by pimonidazole hydrochloride staining or EF5 reduction that occurs at oxygen partial pressure lower than 10 mmHg (1.32%) [5] [6] [7].

As tumor hypoxia contributes to resistance to radio- and chemotherapy and promotes metastasis formation, it represents a major research and therapeutic target. Three-dimensional (3D) multicellular spheroids derived from cancer cells are a particularly appropriate model to investigate oxygen partial pressure gradients and their effects on tumor growth. Indeed, the organization of 3D multicellular tumor spheroids accurately reproduces the heterogeneity of a tumor microdomain with its cell-cell and cell-microenvironment interactions [8–10]. A tumor spheroid and a non-vascularized tumor microdomain are similarly organized and their growth leads to the development of hypoxia and acidosis in their central region. The most external regions of spheroids are exposed to the highest oxygen concentration, like the tumor stroma in close proximity to blood vessels. Conversely, the spheroid inner region mimics the central core of the tumor microdomain, far away from nutrient sources. In a pioneering work published in 1986, dioxygen concentration gradients were measured in large human colon cancer spheroids with a central necrotic core using microelectrodes [11]. It showed that these gradients display a curving profile with a dramatic drop in oxygen partial pressure within the viable rim of cells surrounding the necrotic center. The lowest dioxygen concentration measured inside spheroids smaller than 500 μm in diameter was around 25mmHg. Conversely, in larger spheroids, the central core was necrotic and totally anoxic [11]. These oxygen partial pressure gradients could result from oxygen diffusion and consumption. Recently, a mathematical method has been developed to analyze oxygen consumption rate and to estimate the local partial pressure of oxygen in growing spheroids without need of invasive measurements [12]. This method is based on physical first principles, such as the diffusion equation, and was validated using sections from spheroids derived from the human colon carcinoma cell line DLD1. The data obtained with the model fitted the experimental detection of the hypoxic region in DLD1 spheroids at different stages of growth.

Here, we investigated how growth in physioxia affects the cell proliferation rate and regionalization in 3D multicellular spheroids derived from HCT116 human colon adenocarcinoma cells that were grown at 21% oxygen (the classical concentration used in tissue culture) or 5% oxygen (physioxic condition). We then used the obtained data to mathematically model and compare the oxygen partial pressure gradients in both conditions. We found that oxygen partial pressure is a major rate-limiting factor with a critical effect on cell proliferation rate and regionalization only in spheroids grown in physioxic conditions. Our observations raise many questions on the relevance of the results obtained in “normoxic” (21% oxygen) experimental conditions in which most cancer biology studies and anticancer drug assessments in 3D models are performed.

## Materials and Methods

### Cell Culture Conditions and Spheroid Generation

HCT116 colon adenocarcinoma cells (ATCC) were cultured in DMEM (Invitrogen) containing 10% FCS with 2mM/l glutamine and penicillin/streptomycin in a humidified atmosphere of 5% CO_2_ at 37°C. Spheroids were prepared as previously described [13]. Briefly, 500 cells/well were distributed in ultra-low attachment 96-round bottom well plates. Plates were centrifuged (200g for six min) and then placed in a humidified atmosphere of 5% CO_2_ at 37°C. Cells and spheroids were cultured under atmospheric oxygen concentration, or in regulated hypoxia confinement (5% oxygen) by using an hypoxia workstation at the EFS French Blood Institute hypoxia facility, Toulouse. Spheroid volume was obtained by manually measuring two orthogonal diameters, d1 and d2, using an inverted microscope maintained in the hypoxia workstation. Spheroid volume was calculated according to the formula: V = 4πd1^2^d2/3.

### Spheroid Cryosections

Spheroids were fixed in formalin (Sigma) for 2–3 hours, then washed with PBS and stored at 4°C. After fixation, spheroids were incubated in 15% and then 30% sucrose in PBS at 4°C for 24 h, embedded in Tissue-Tek (Sakura Finetek) and 5μm cryosections were cut.

### EdU Labeling of Spheroids

EdU labeling was performed by using the Click-it® EdU Alexa Fluor® Imaging Kit (Molecular Probes). Briefly, EdU was added to the culture medium at a final concentration of 10μM. After 24h incubation, spheroids were rinsed in PBS and fixed. EdU detection, based on a “click” reaction between EdU and the Alexa FLuor® 488 dye, was performed following the manufacturer's instructions.

### Hypoxia Detection

Hypoxia was detected using the Hypoxyprobe™-1 Plus Kit (HPI). Briefly, pimonidazole hypochloride (Hypoxyprobe™-1) was added to the culture medium at a final concentration of 100μM at 37°C for 2h. In hypoxic cells, pimonidazole hypochloride forms stable adducts with proteins. After spheroid fixation, pimonidazole adducts were detected by incubating cryosections with FITC-conjugated MAb1 (the monoclonal antibody provided in the kit, 1/300) at 37°C for 2h. After PBS washes, DNA was stained with DAPI.

### Image Acquisition and Analysis

Transmitted light images of spheroids were acquired using a routine microscope. Fluorescence images of 5μm spheroid cryosections were acquired using a DM5000 (Leica) epifluorescence microscope, fitted with a Roper COOLsnap ES CCD camera. Images were processed using the Metavue and ImageJ software packages. Before automated analysis, images with artefacts were manually corrected. Specifically, parts of other spheroids, due to the sectioning process, or staining background and debris were removed using the clearing function of Image J.

### Modeling the Spheroid Oxygen Partial Pressure Profile

The spheroid oxygen partial pressure profile was modeled by using a previously published diffusion model that takes into account oxygen diffusion and consumption within spheroids [12]. This mathematical model is based on a diffusion equation (with the hypothesis that the partial pressure at the center of the spheroid is zero) and the fully analytical form of the model requires experimental data (spheroid radius, anoxic radius and proliferative layer, which is the difference between the two previous values). In this model, oxygen consumption rate is assumed to be constant; however, a deeper analysis suggests only minor differences in prediction if a non-constant oxygen consumption is assumed [14].

## Results

### 3D Spheroid Growth Is Reduced in Physioxic Culture Condition

**Fig 1:**
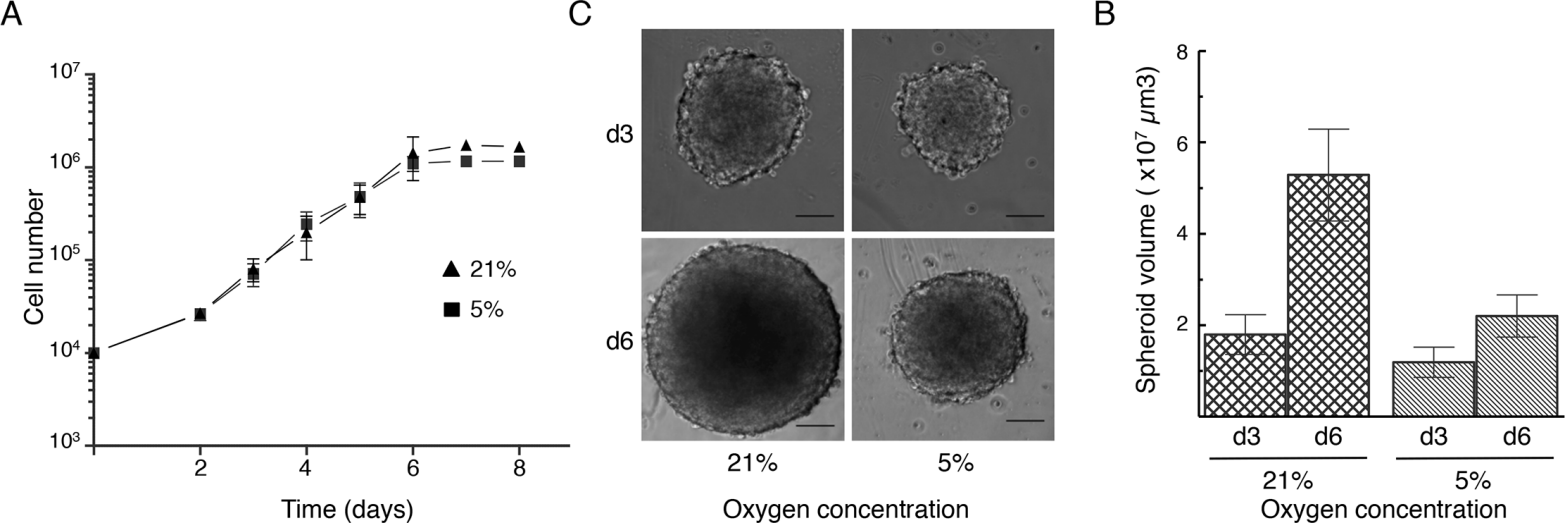
Effect of oxygen concentration reduction on the proliferation of 2D monolayers and 3D spheroids derived from HCT116 cells. (A) Growth curve of HCT116 cells grown in the presence of 21% or 5% oxygen. These data are the mean ± SD of four independent experiments. (B,C) Spheroids prepared from monolayers of HCT116 cells maintained at the targeted oxygen concentration (21% or 5%) were grown in 96-well plates. (B) Representative bright field microscopy images of spheroids grown at the indicated oxygen concentrations for three and six days. Scale bar is 100 μm. (C) Spheroids volume was determined after three and six days (d3 and d6) of culture. Results are the mean ± SD of the measurements made in five independent experiments: n = 88 spheroids (d3, 21%) n = 57 (d6, 21%), n = 80 (d3, 5%), n = 50 (d6, 5%).

To avoid any effect associated with an acute reduction of oxygen concentration, HCT116 cells grown as monolayers and used to prepare 3D spheroids were continually maintained at 21% (considered as normoxia condition) or at 5% oxygen concentration. In both conditions, cell proliferation rates were comparable with a calculated doubling time of about 18 hours (Fig 1A). This indicates that an oxygen concentration of 5% (in otherwise similar and optimal environmental conditions) is sufficient to ensure the optimal proliferation of cells grown as monolayers.

Multicellular spheroids were then prepared by cell aggregation on low-attachment 96-wells plates (see Methods) and cultured at 5% or 21% oxygen concentration, like the cell monolayer from which they were derived. Bright field microscopy observation of representative spheroids for each experimental condition at day 3 and 6 of culture (Fig 1B) suggested that growth was inhibited by culture at 5% oxygen. This observation was confirmed by microscopic measurement of the spheroid size prior to fixation in the controlled atmosphere zone at day 3 and 6 after seeding (Fig 1C). Spheroids grown at 21% oxygen concentration reached 500 μm in diameter after six days. Conversely, already after three days of culture, the size of spheroids cultured at 5% oxygen concentration was smaller than that of spheroids grown in normoxic conditions and this difference became even more apparent at day 6. Moreover, spheroid growth was inhibited also when spheroids cultured at 21% oxygen concentration for three days were transferred to the low oxygen concentration, or when cells cultured at 21% oxygen were used to form spheroids in physioxic condition (data not shown).

Furthermore, the absence of C-PARP cleavage detection by immunofluorescence on cryosections indicated that apoptosis was not induced when 3D spheroids were grown up to 6 days at 5% oxygen concentration (data not shown).

Such a dramatic growth reduction in 3D cell spheroids grown at 5% oxygen, without any obvious difference in 2D monolayer cultures, suggests that this low oxygen concentration might affect the cell proliferation rate and regionalization due to limited oxygen diffusion within spheroids.

### Proliferation Is Restricted in Spheroid Grown in Physioxic Condition

**Fig 2:**
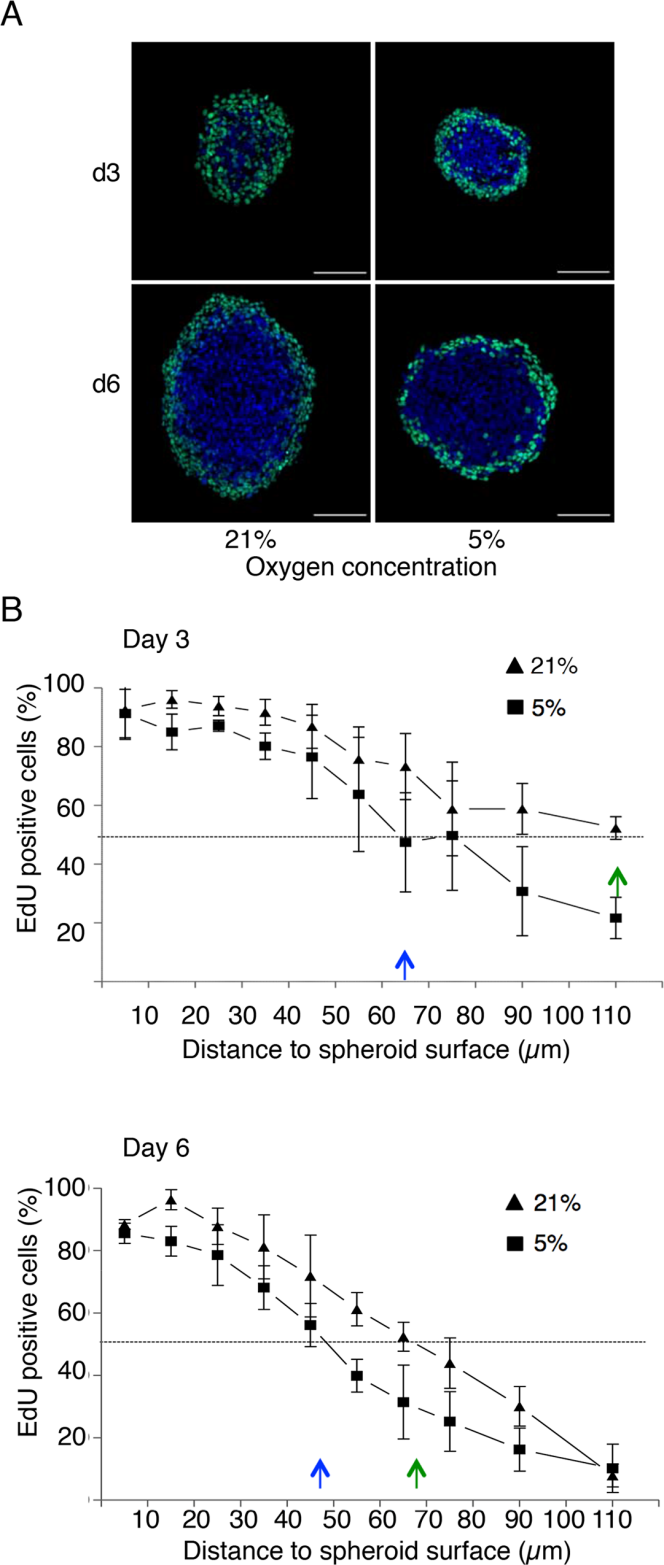
Cell proliferation regionalization in 3D spheroids grown at 5% and 21% oxygen. HCT116 spheroids grown in the indicated conditions for three (d3) and six days (d6) were incubated with EdU for 24 hours prior to fixation and analysis. (A) Representative fluorescence microscopy images of cryosections from spheroids grown at the indicated oxygen concentrations for three and six days and labeled with EdU (green) and DAPI (blue). Scale bar, 100 μm. (B) Percentage of EdU-positive cells relative to the distance from the spheroid surface. The results are the mean +/-SD of five independent experiments with 4 to 5 spheroids analyzed per condition in each experiment. Data were extracted from the automated segmentation and quantification of images of cryosections cut along the spheroid center (see Methods). Arrows (blue: 5%, green: 21% oxygen) indicate the In Depth Proliferation 50% index (IDP_50_) that is the distance from the surface where 50% of cells are EdU-positive.

Spheroid growth inhibition in physioxic condition could result from a reduction of the proliferation rate at the spheroid periphery and/or from a steeper proliferation gradient in the most central part of the spheroids. To quantify the proportion of proliferating cells as a function of the distance from the spheroid surface at day 3 and 6 of culture in normoxic and physioxic conditions, spheroids were incubated with EdU for 24 hours to label cells committed to cell division or that have undergone replication [13]. As previously reported, at day 3 at 21% oxygen, EdU was homogeneously incorporated in most of the spheroid volume, indicating that the whole cell population was actively proliferating (Fig 2A). At day 6, proliferation was restricted to the most external spheroid layers due to the establishment of a proliferation gradient [13]. Conversely, in spheroids grown at 5% oxygen, proliferation was mostly restricted to the most external layers already at day 3 and was clearly limited to a narrow external layer at day 6 (Fig 2A).

To quantify precisely these observations, cryosections from spheroids grown in the two experimental conditions (see legend to Fig 2 for details on sample numbers) were processed through a pipeline for automatic image acquisition, nucleus segmentation and determination of EdU positivity. The results (percentage of EdU-positive cells) were then analyzed considering the distance of each nucleus from the spheroid surface (Fig 2B). These data confirmed the visual observations presented in Fig 2A and indicated that the percentage of proliferative cells in the outermost cell layer (10μm of depth) at day 3 and 6 was similar in both conditions. A proliferation gradient was visible already at day 3 in physioxic condition, while it was less pronounced for spheroids grown in normoxic condition. To characterize this gradient, the In Depth Proliferation 50% index (IDP_50_) was used. This new index represents the distance from the surface where less than 50% of cells are proliferating (*i*.*e*., EdU-positive). At day 3, the IDP_50_ was 55 μm for spheroids grown at 5% oxygen (blue arrow in Fig 2B) and 110 μm for spheroids at 21% oxygen (green arrow). At day 6, the two IDP_50_ values dropped to 45 μm and 70 μm (5% and 21% oxygen concentration, respectively). This indicates that, although a proliferation gradient is observed also at 21% oxygen concentration, culture at 5% oxygen concentration has a stronger inhibitory effect on spheroid growth with a very limited layer of proliferating cells.

These data demonstrate that in 3D spheroids, physioxia induces a steep proliferation gradient, associated with an earlier regionalization of proliferation to a very thin external cell layer. This suggests the existence of an oxygen rate-limiting threshold that leads to cell cycle arrest.

### Detection of the Spheroid Hypoxic Core

**Fig 3:**
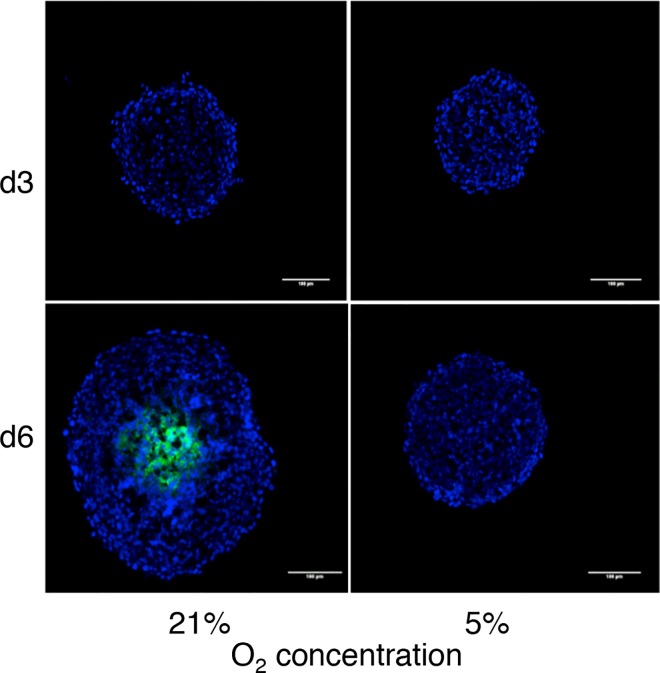
Detection of hypoxia with pimonidazole hypochloride. HCT116 spheroid grown in the indicated conditions (5% and 21% oxygen) for three (d3) and six days (d6) were incubated with pimonidazole hypochloride for 2h. Pimonidazole-positive hypoxic cells were detected in spheroid cryosections with the FITC-conjugated MAb1. Nuclei were stained with DAPI. Representative fluorescence microscopy images of spheroids at day 3 and 6. Pimonidazole-positive hypoxic cells are in green and nuclei in blue. Scale bar, 100 μm.

To further investigate this question, hypoxia occurrence in physioxic and normoxic conditions was monitored by incubating spheroids with pimonidazole hypochloride. Labeling occurs when oxygen concentration is below 10mmHg (1.32%). No pimonidazole staining was detected at day 3 (both at 5% and 21% oxygen) (Fig 3A). At day 6 pimonidazole staining was rarely detected in spheroids grown at 5% oxygen, while a strong staining was observed in the core of spheroids grown at 21% oxygen concentration.

To determine the distance of the pimonidazole-positive area from the spheroid surface, the intensity of each pixel was extracted and plotted relative to its distance from the surface. In spheroids grown at 21% oxygen for six days, the average distance of pimonidazole-positive cells from the spheroid surface was about 143 μm. Conversely, no pimonidazole-positive cell was detected in spheroids grown at 5% oxygen at day 6, suggesting that they have not reached a sufficient diameter for the available oxygen concentration to fall below 1.32% at their center.

### Modeling the Oxygen Partial Pressure Profile in Spheroids at Day 6 of Culture

**Fig 4:**
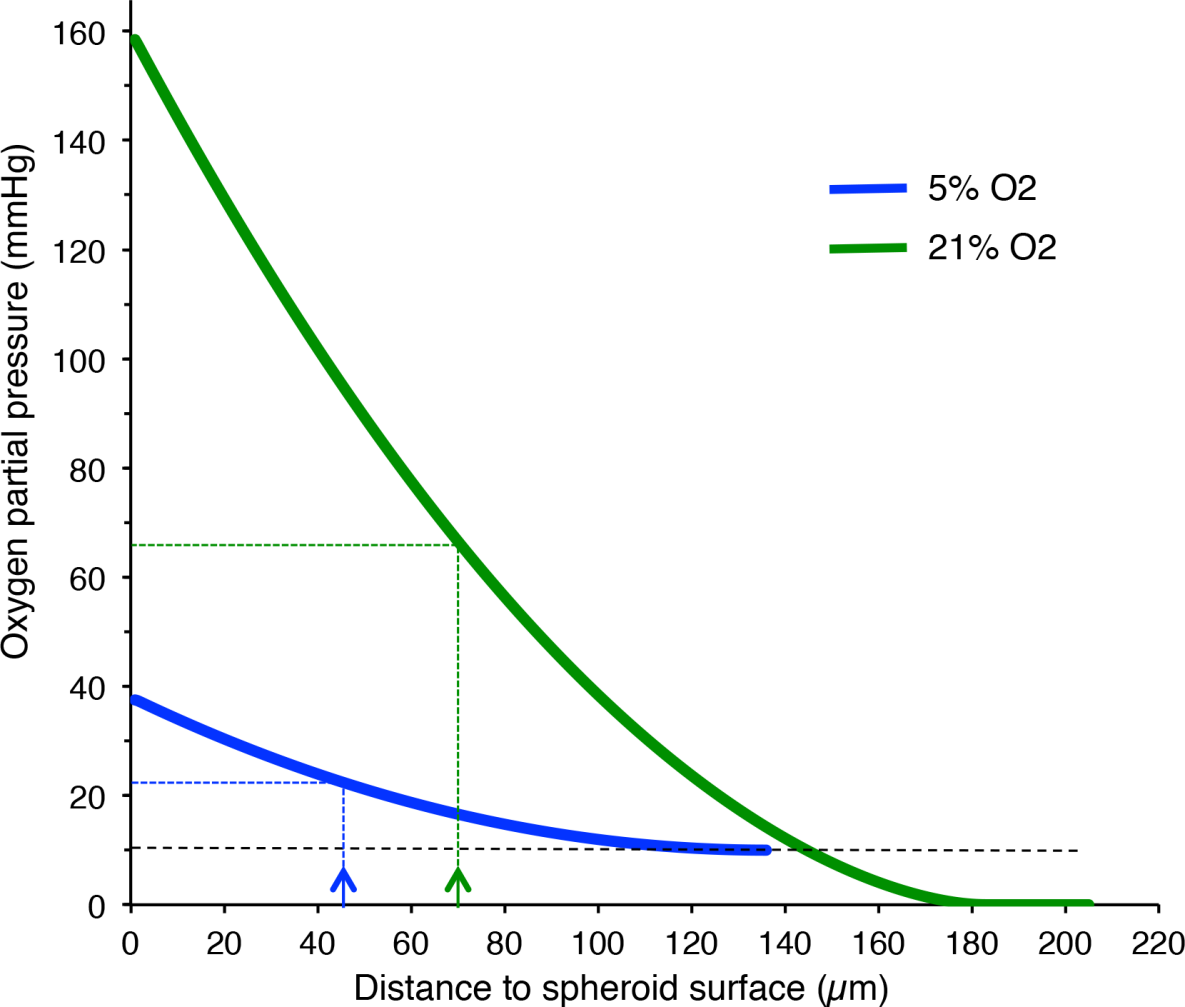
Simulated oxygen partial pressure profiles in 3D spheroids. Simulation of the oxygen partial pressure profile after six days of growth at 5% (blue) and 21% (green) oxygen according to the model proposed in [12]. The blue and green arrows and the dotted lines indicate the IDP_50_ at 5% and 21%, respectively (data from Fig 2).

To further explore this hypothesis and in the absence of an adequate experimental approach to directly measure oxygen partial pressure within microtissues, the oxygen partial pressure profile within a spheroid was modeled by using a previously published diffusion model [12]. Determination of the distance from the surface of pimonidazole-positive cells in spheroids grown in 21% oxygen allowed setting the 10mmHg limit at about 143μm from the spheroid surface. The absence of detectable pimonidazole-positive cells at day 6 in spheroids grown in 5% oxygen indicates that oxygen partial pressure at the center of such spheroids was at least 10mmHg. We therefore hypothesized that the oxygen concentration at the center of these spheroids was nearly 10 mmHg. Fig 4 shows the simulated oxygen partial pressure profiles generated using these data relative to the distance from the spheroid surface at the two oxygen concentration conditions. These curves indicated that, in normoxic culture conditions, the IDP50 (70 μm) corresponded to a reduction of the oxygen partial pressure from 160mmHg to 65mmHg. In physioxic conditions, the IDP50 (45 μm) corresponded to a reduction of the oxygen partial pressure from 40 mmHg to 25mmHg.

## Discussion

In this work we used a 3D tumor spheroid model to investigate how reduction of environmental oxygen to a concentration that is comparable with tissue normoxia affects cell proliferation and regionalization in a microtissue.

Growth of HCT116 human colon adenocarcinoma cells in monolayers was not obviously modified when the oxygen concentration in the medium was lowered from 21% to 5%. Thus, when cells are grown as monolayers, a 5% oxygen concentration provides sufficient oxygen to ensure cell division and to sustain proliferation. This result also suggests that when a tissue is exposed to such low oxygen concentration, cells located at the tissue surface or in the immediate vicinity proliferate optimally. Indeed, this is confirmed by the observation that, in 3D spheroids, cells at the spheroid surface proliferate well both at 5% and 21% oxygen concentration. On the other hand, 3D spheroid growth was strongly inhibited when oxygen concentration was reduced to 5%, compared with spheroids cultured at 21% oxygen. However, as 5% oxygen is the physiological concentration in tissues, one should not talk about growth inhibition in physioxic culture conditions, but rather of growth stimulation upon exposure to the non-physiological 21% oxygen concentration.

To characterize the regionalization of cell proliferation in 3D spheroids and to quantify how proliferation parameters are progressively modified within the spheroid volume, we used the In Depth Proliferation 50% index (IDP_50_) that represents the distance from the surface where only 50% of cells are still cycling (EdU-positive). We found that after six days of growth, IDP_50_ is approximately 45μm in spheroids grown in 5% and 70μm in spheroids cultured at 21% oxygen. This suggests that a lower environmental oxygen concentration results in a faster decrease of the oxygen available within the spheroid, leading to cell cycle arrest. Again, this conclusion could be rephrased by saying that increasing to 21% the external oxygen concentration results in higher oxygen partial pressure deeper in the spheroid, thus keeping cells, that otherwise would be quiescent, in contact with an oxygen level that is sufficient to sustain proliferation.

In agreement, modeling the oxygen partial pressure profiles, using a mathematical model developed to analyze oxygen consumption and to estimate the local partial pressure in growing spheroids [12], showed that at 5% oxygen, the IDP_50,_ (about 45μm) (see blue arrow in Fig 4) corresponds to a partial pressure of 25 mmHg. This means that at 5% oxygen, proliferation is fully ensured only in the utmost layers of spheroids grown and in 2D monolayers. These observations suggest that oxygen partial pressure is a major rate-limiting parameter in spheroid grown at 5% oxygen with a 20mmHg threshold that is required to sustain proliferation and below which 50% inhibition of proliferation is observed. In contrast, when spheroids are grown at the non-physiological 21% oxygen concentration, proliferation is artificially sustained further away from the spheroid surface and is progressively reduced by the limited diffusion of other parameters. One can speculate that specific nutrients and growth factors might be the limiting factors, however their identification and their incorporation in the modeling system will require further investigation.

In vivo, microdomains of malignant solid tumors are surrounded by an extracellular matrix that comprises various cell types (fibroblasts, immune cells) and capillary vessels. The oxygen concentration in these terminal vessels is thought to be in the range of 5%-8% depending on the tissue type and the local consumption by fibroblasts and immune cells will decrease the available oxygen at the edge of the tumor microdomain. Several publications (see [15] and references therein) have reported that pimonidazole positivity in microtumor cords is detected about 100 μm away from blood vessels and is associated with cell cycle arrest. Our results are fully in agreement with these observations that were made by pathologists and that validate the use of 3D cancer cell spheroids as a microtumor model.

It has been proposed that low oxygen concentrations still allow the proliferation, but decrease the differentiation rate of stem cell populations. Cell division in limited oxygen concentration conditions would thus ensure the self-renewal and maintenance of the stem cell population, without any commitment to differentiation (reviewed in [1]). Our findings strengthen the need to re-explore basic cancer cell biology issues in physioxia and not in artificial hyperoxia (21% oxygen concentration).

Thanks to the recent technical developments in cancer cell spheroid production and to the progress in understanding their biology [9, 16, 17], this preclinical model is becoming an essential step in the development of anti-cancer strategies [18]. For instance, at low oxygen concentrations, sensitivity to radiation therapy is reduced in spheroids, as observed in tumors. Similar findings were made concerning the activity of therapeutic agents [10, 19]. This is because oxygen is a potent radio-sensitizer. Indeed, regions with high oxygenation (>20mmHg) are up to three times more radiosensitive than anoxic areas. This phenomenon is known as the oxygen enhancement ratio [20]. Thus, our results urge to consider that, for physiological relevance, the evaluation of anticancer drugs in 3D microtissues should be performed in physioxic conditions.
